# Development and characterisation of a high-sensitivity X-ray CT polymer gel dosimeter

**DOI:** 10.1007/s13246-025-01586-2

**Published:** 2025-07-09

**Authors:** Jonathon Lexor Lumley, Pejman Rowshanfarzad, Mounir Ibrahim, Mario Djukelic, David J. Henry

**Affiliations:** 1 School of Physics, Mathematics and Computing, University of Western Australia, Crawley, WA Australia; 2 Department of Medical Technology and Physics, Sir Charles Gairdner Hospital, Hospital Avenue, Nedlands, WA Australia; 3 Centre for Advanced Technologies in Cancer Research (CATCR), Perth, WA Australia; 4 Department of Radiation Oncology, Sir Charles Gairdner Hospital, Nedlands, WA Australia; 5 Chemistry and Physics, College of Science, Health, Engineering and Education, Murdoch University, Murdoch, WA Australia

**Keywords:** Polymer gel dosimetry, X-ray CT, Optimisation, PASSAG-N, Sensitivity

## Abstract

Polymer gel dosimeters have shown potential for clinical 3D dosimetry; however, their use has been limited due to low sensitivity and reliance on scarcely available magnetic resonance imaging. This study aimed to optimise a PASSAG (Poly AMPS Sodium Salt And Gelatin) polymer gel dosimeter for X-ray computed tomography, to enhance its clinical feasibility. The total monomer concentration was increased to improve sensitivity, and different cosolvents were tested to enhance the limited solubility of N, N’-methylenebisacrylamide, the crosslinker. n-propanol was identified as the optimal cosolvent, allowing for an 18.3% monomer concentration, 30% crosslinker to total comonomer mass gel, at a 3:7 cosolvent-to-water ratio. The optimised formulation, PASSAG-N (PASSAG– n-propanol), consisted of 54.4% w/w deionised water, 23.3% n-propanol, 12.8% 2-acrylamido-2-methylpropane sulfonic acid sodium salt, 5.5% N, N’-methylenebisacrylamide, 4.0% gelatin, and 0.089% (4.65mM) tetrakis (hydroxymethyl)phosphonium chloride. The dosimeter was irradiated within a standard timeframe to assess its sensitivity, and theoretical calculations confirmed its equivalence to water, soft tissue, brain, and muscle. Compared to a cosolvent-free formulation, PASSAG-N exhibited a 250% increase in Hounsfield unit (HU) change, demonstrating enhanced sensitivity. The optimised gel showed a linear response over a 1–12 Gy dose range, with an average sensitivity of 1.072 ± 0.041 HU Gy⁻¹ and a dose resolution ≤ 0.31 Gy, making it a promising alternative for clinical X-ray computed tomography-based dosimetry. This study highlights the potential of PASSAG-N as a highly sensitive and potentially practical polymer gel dosimeter for clinical applications.

## Introduction

The 21st century has seen a steady increase in the conformity of radiotherapy techniques, with the introduction of intensity modulated radiation therapy, volumetric modulated arc therapy, stereotactic ablative radiation therapy, etc. The increase in conformity has also led to an increase in complexity, with a need to develop suitable dosimeters to allow for further optimised treatment plans, whilst upholding a certain level of accuracy and confidence during treatment deliveries [1]. With the inclusion of small field sizes becoming ever more present in treatment plans, the use of conventional dosimeters can lead to a myriad of problems resulting in inaccuracies [2]. Polymer gel dosimeters (PGDs) could be useful for small-field dosimetry because they eliminate problems associated with volume averaging, tissue equivalency, directional dependency, and complex media corrections [2]. Furthermore, unlike conventional dosimeters, PGDs can be contoured to anthropomorphic shapes and generate truly inherent 3D dose distributions, providing unparalleled 3D spatial resolution required for complex radiotherapy plans [3, 4]. These gels work by utilising radiolysis-induced polymerisation to capture dosimetric information that is proportional to the absorbed dose. The polymerised molecules have different characteristics from their monomer counterparts, which can be exploited through various imaging modalities [4]. Currently the gold standard for PGD imaging is magnetic resonance imaging (MRI). However, when considering clinical implementation, this modality requires a considerable amount of imaging time and introduces additional environmental dependences [5]. Due to short scanning times, excellent image uniformity and accessibility relative to MRI, the use of X-ray computed tomography (CT) for PGD imaging provides many advantages [5, 6]. Currently the largest obstacle for X-ray CT imaged PGDs is a lack of sensitivity and dose resolution [4]. PGD imaging can also be conducted using optical computed tomography (OCT), first implemented by Gore et al. utilising the change in transparency of gel as its irradiated [7]. Similarly to X-ray CT, OCT is not plagued by significant temperature dependencies seen in MRI [8], and is viewed as a low-cost alternative to X-ray CT. Further problems surrounding clinical PGD implementation are the large variety of new gel formulations being tested with relatively little emphasis placed on optimising pre-existing formulations. One approach to optimise pre-existing PGDs has been through the addition of co-solvents. Using a 30% cosolvent-to-water ratio, Koeva et al. developed a 10%T 50%C N-isopropylacrylamide based NIPAM dosimeter with a sensitivity of 0.399 HU Gy^-1^, a 37.1% increase compared to the original 6%T, 50%C formulation [9]. Chain et al. took a different approach, using the NIPAM monomer itself as a cosolvent to create a 19.5%T 23%C gel with a linear response up to 19 Gy, along with a sensitivity and dose resolution of 0.88 HU Gy^-1^ and ≤ 0.20 Gy respectively [10]. All current PGDs have undesirable characteristics, complicating their implementation into a clinical environment. Despite demonstrating better dosimetric properties, anoxic PGDs require complex manufacturing procedures and laboratory expertise that is not commonly available in a radiation oncology department. Acrylamide based PGDs such as PAG and PAGAT are highly toxic with acrylamide being both teratogenic and carcinogenic [11]. Whilst being less toxic, the NIPAM dosimeter exhibits lower sensitivity relative to other formulations [9, 12]. Methacrylic acid based PGDs such as MAGAT exhibit relatively high sensitivity [13], however, are observed to be significantly dose rate dependent [14, 15]. Recently Farhood et al. investigated using a 2-acrylamido-2-methylpropane sulfonic acid sodium salt-based monomer (named PASSAG) which showed good sensitivity when imaged with MRI, whilst having significantly reduced monomer toxicity [11]. Further studies showed the normoxic PASSAG had a considerable number of ideal characteristics when compared to other tested PGDs, displaying both beam energy and dose rate independence whilst having adequate temporal stability [16]. The aim of this paper was to optimise the composition of PASSAG, to maximise its sensitivity and thereby make it more feasible for X-ray CT imaging. Theoretical tests were also carried out to determine the effect optimisation had on tissue equivalence.

## Methods

### Fabrication

The general dosimeter fabrication used in this study is described below; however, the concentrations of water, cosolvent, monomer, and crosslinker were subject to various optimisation tests and are discussed in the subsequent composition optimisation section. The PGDs were manufactured inside a fume hood under normal atmospheric conditions.

The general procedure for the preparation of the PGDs was as follows. Room temperature de-ionised (DI) water and cosolvent (if used) were added to a beaker, followed by 4% w/w gelatin (300 Bloom Type A, Sigma-Aldrich), which was left undisturbed for five minutes to allow for the blooming of the gelatin. It was important to add the gelatin after the cosolvent, as this resulted in less coagulated protein chains and produced an overall stronger gel product, especially at higher cosolvent concentrations. After the blooming period had elapsed, N,N’-methylenebisacrylamide (BIS) (99%, Sigma-Aldrich) was added, and the mixture was heated to 40 ^o^C to allow for faster dissolution. Once fully dissolved, 2-acrylamido-2-methylpropane sulfonic acid (AMPS) sodium salt solution (50% wt in H_2_O, Sigma-Aldrich) and 4.65 mM of tetrakis (hydroxymethyl)phosphonium chloride (THPC) (80% in H_2_O, Sigma-Aldrich) were introduced. The solution was stirred for an additional minute to ensure a homogenous distribution before being poured into 20 ml high-density polyethylene liquid scintillation vials (27 × 51 × 1 mm, PerkinElmer, USA). Each vial was wrapped in parafilm and aluminium foil to minimise oxygen penetration and prevent photo-polymerisation, before being stored at 5 ^o^C for one hour to allow the gel to set.

### Irradiation

**Fig. 1:**
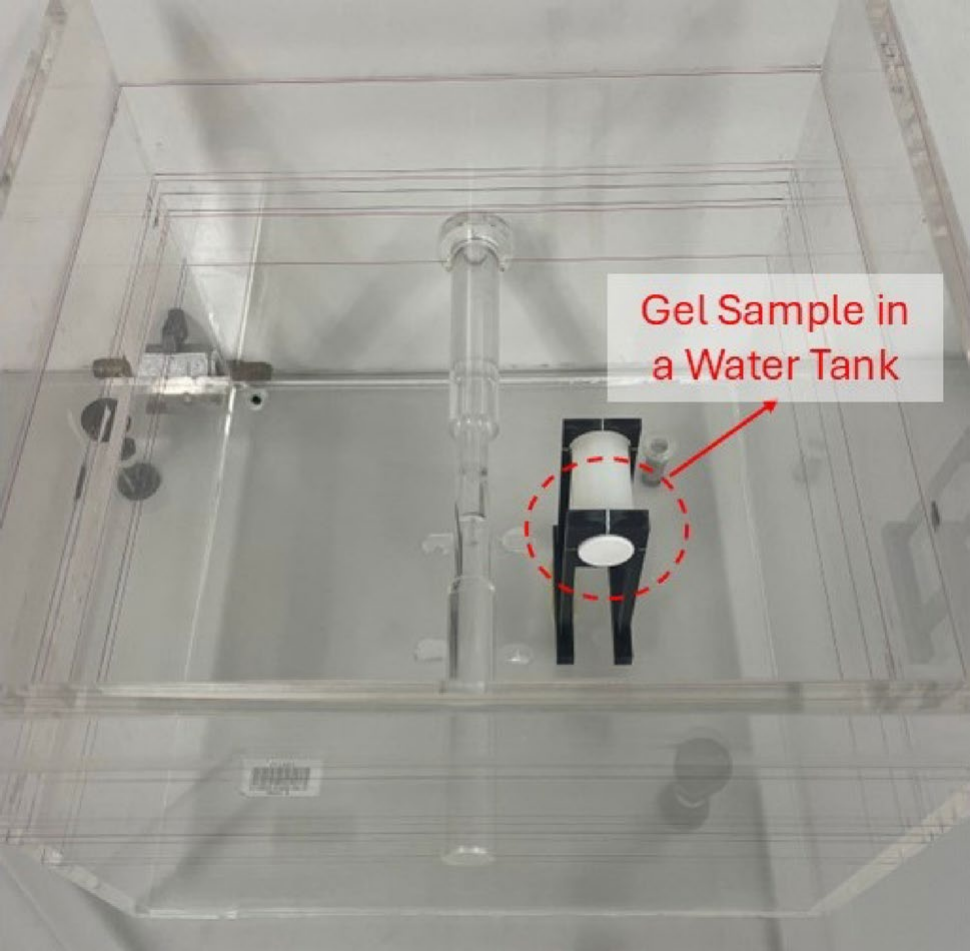
Water bath setup used to precisely irradiate each gel vial individually and account for photon scatter

Irradiations were conducted using 6 MV photon beams generated by a TrueBeam linac (Varian Medical System, USA). As shown in Fig. 1, the vials were individually placed in a 30 × 30 × 25 cm^3^ water bath and suspended 10 cm above the base of the bath using a 3D-printed polylactic acid (PLA) mount to achieve adequate photon backscatter. The vials were positioned with their elongated axes perpendicular to the central axis of the beam. The water bath was filled 10 cm above the midpoint of the vial to allow for buildup and further photon lateral scatter. A source-to-skin distance of 90 cm to the water surface was used, with a 10 × 10 cm^2^ field size and a 600 MU/min dose rate. The linac was calibrated to deliver 1 MU per 1 cGy at d_max_ with a 10 × 10 cm² field size using the isocentric technique. The delivered dose at a depth of 10 cm was adjusted using the tissue maximum ratio (TMR) measured factor.

A 180° rotation was applied halfway through the irradiation process to ensure a uniform dose distribution. Immediately after irradiation, each vial was kept in a dark, room-temperature cooler bag to minimise effects from potential environmental dependencies.

### Imaging

All X-ray CT images were captured using a Siemens Somaton Definition AS+ CT scanner (Siemens, Germany). Images were acquired at 140 kV and 300 mAs, following recommendations made by Hilts [17]. Imaging parameters included using a standard head routine axial scan with a 1.0 s rotation time, 5.0 mm reconstructed slice thickness, a total collimation width of 38.4 mm (32 × 1.2 mm), and an H31s (standard head) reconstruction kernel. Four images of both irradiated and unirradiated samples were acquired for background subtraction and image averaging, unless stated otherwise. Imaging averaging was conducted to increase the signal-to-noise ratio and remove image artefacts. Although not as effective as, for example, a 25-image average, a four-image average was chosen to reflect practical imaging times in a busy radiation oncology department. A scanner warm-up sequence was performed prior to each imaging session to minimise X-ray tube temperature-induced variations in response [6]. Up to twelve vials were scanned simultaneously, with their long axis positioned perpendicular to the central axis of the X-ray beam. Using a low-density 200 × 120 mm polystyrene cylinder, vials were placed in a circular arrangement to uniformly distribute any absorbed dose.

### Processing

Image processing was carried out using MATLAB (Mathworks, Natick, MA USA). Averaged unirradiated images were subtracted from averaged irradiated images to produce a background-subtracted image. A 2D Weiner filter (7 × 7 span, singular iteration) followed by a remnant artifact removal filter, developed by Jirasek et al. [18], (window size = 5, polynomial degree = 1, iterations = 4) was then applied to minimise image noise. Hounsfield units (HU) of each vial were obtained from the mean grey scale values using ImageJ image processing software (National Institutes of Health, USA). Circular regions of interest with a 30-pixel diameter were placed at the centre of each gel position. The same-size regions of interest were uesd throughout all measurements to ensure consistency in HU determination. All data analysis and graphing were conducted using OriginPro (OriginLab, USA).

### Composition optimisation

#### Optimal cosolvent determination

Five cosolvents were tested to improve BIS solubility; methanol, ethanol, n-propanol, isopropanol and t-butanol. A gravimetric method was used to determine the BIS solubility in each cosolvent. 15% w/v of BIS was added to a 10 ml solution containing a 3:7 w/w ratio of cosolvent-to-water to ensure saturation. The crosslinker was allowed to dissolve in solution for 1.5 h at 18.5 ^o^C, occasionally being stirred to ensure uniform dispersity. Each vial was sealed with parafilm and manually stirred to prevent a supersaturate from forming. The saturated solution was then filtered into a pre-weighed watchglass (m_container_), quickly weighed (m_solution_), then heated to 100^o^C to evaporate the solvent. The watchglass was removed and weighed (m_solute_) at hourly intervals until no mass change was observed. The experiment was repeated three times with the solubility of BIS in each cosolvent calculated using Eq. 1.

1 $${\%\text{w}}/{\text{w}}_{{{\text{solute}}}} = \frac{{{\text{m}}_{{{\text{solute}}}} - {\text{m}}_{{{\text{container}}}} }}{{{\text{m}}_{{{\text{solution}}}} - {\text{m}}_{{{\text{container}}}} }} \times 100$$

#### Optimising the cosolvent ratio

To determine which cosolvent-to-water ratio would exhibit the highest sensitivity, employing the previously described gravimetric method, five cosolvent-to-water ratios were tested, 0:10, 1:9, 2:8, 3:7 and 4:6. Determination of C_max_ (the maximum amount of crosslinker dissolvable in solution) was required as it is the limiting factor in creating high %T (total monomer concentration) formulations. The total % w/w of a gel formulation can be expressed by Eq. 2.

2 $$Total\:gel\:{\%}_{w/w}=S+M+C+G=100$$

Where S is the % w/w of the water-cosolvent solvent; M is the % w/w of the monomer; C is the % w/w of the crosslinker and G is the % w/w of gelatin. For a gel formulation consisting of 30%C (crosslinker to total comonomer mass), M can be rewritten as shown in Eq. 3.

3 $$M=7C/3$$

The amount of water-cosolvent solvent required to completely dissolve the BIS depended on the crosslinker's solubility limit (L) in each cosolvent-to-water ratio, as described in Eq. 4.

4 $$S=C/L$$

Substituting Eqs. 3 and 4 back into Eq. 2 allows C_max_ to be determined for a known % w/w gelatin recipe using Eq. 5, thereby maximising %T. Once C_max_ is known, both M and S could be back-calculated using Eqs. 3 and 4, respectively.

$$\frac{C}{L}+\frac{7C}{3}+C+G=100$$

$$\:\Rightarrow\frac{1}{L}+\frac{10}{3}=\frac{(100-G)}{C}$$

5 $$\:\Rightarrow\:C_{max}=\frac{(100-G)}{(\frac{10}{3})+(\frac{1}{L})}$$

Vials were irradiated approximately 2 h post-manufacture, receiving doses of either 1 or10 Gy to represent low and high dose regions, respectively. Imaging was conducted 24 h post-irradiation.

To determine whether the addition of the co-solvent had any direct effect on the degree of polymerisation within the gels, a series of gels were prepared with varying amounts of cosolvent, based on the water-to-cosolvent ratios mentioned above. The amount of monomer and crosslinker was kept identical for each gel sample (based on the crosslinker solubility in DI water). This test removed any dependencies on %T, ensuring that any changes in the degree of polymerisation, measured through ΔHU, were a direct result of co-solvent addition. All other fabrication, irradiation and imaging methods were kept identical to the description above.

### Theoretical determination of tissue equivalency

The density of the unirradiated gel (ρ_0_) was determined using the method described by Trapp et al. [19]. Measurements were conducted at 18.5 ^o^C and averaged over three trials. Post-irradiated gel density (ρ_1_) was theoretically determined using the method described by Trapp et al. [20]. ΔHU measurements from two gel batches were taken over the optimised dosimeter’s entire quasi-linear dose range and averaged. Z_eff_ (effective atomic number) and N_e_ (electron density) were calculated using Eqs. 6 and 7, respectively, originally derived by Manohara et al. [21], where f_i_ is the molar fraction of the i^th^ constituent element; (µ/ρ)_i_ is the mass attenuation coefficient of the i^th^ constituent element (calculated through the National Institute of Standards and Technology’s XCOM database); A_i_ is the mass number of the i^th^ constituent element; Z_i_ is the atomic number of the i^th^ constituent element; < A > is the average atomic mass of the gel, and N_A_ is Avogadro’s number.

6 $$ {\text{Z}}_{{{\text{eff}}}} = \frac{{\mathop \sum \nolimits_{{\text{i}}} {\text{f}}_{{\text{i}}} {\text{A}}_{{\text{i}}} \left( {\frac{\mu }{\rho }} \right)_{{\text{i}}} }}{{\mathop \sum \nolimits_{{\text{i}}} {\text{f}}_{{\text{i}}} \frac{{A_{i} }}{{Z_{i} }}\left( {\frac{\mu }{\rho }} \right)_{{\text{i}}} }} $$

7 $${\text{N}}_{{\text{e}}} = \frac{{{\text{N}}_{{\text{A}}} {\text{Z}}_{{{\text{eff}}}} }}{{\left\langle {\text{A}} \right\rangle }} $$

Additionally, the equivalency calculations factored in the presence of THPC, despite only contributing a very small part to the overall elemental composition. The % w/w of THPC added was dependent on the quantity of gel produced, and as such, all calculations were based on a 1.0 kg gel batch. Given that Type A porcine skin gelatin is composed of 19 amino acids [22], an approximation of its elemental composition was used based on the work by C.R Smith [23]. The elemental composition of soft tissue was adopted from the ICRU report 46 [24].

### Sensitivity, dose resolution and dose range determination

To determine the dose range of the optimised formulations, two batches were irradiated between 3- and 4-hours post-manufacture and subsequently imaged 41 h post-irradiation. Samples from each batch were subjected to 0.5, 1, 2, 5, 7, 10, 12 and 15 Gy to produce a dose-response curve, characterised by a second-degree polynomial fit. To determine the sensitivity, a linear fit was employed over the region displaying a quasi-linear response, with the sensitivity derived from the slope of the fit. The background N_CT_ was subtracted to display ΔHU as a function of absorbed dose. Dose resolutions for both batches were calculated using Eq. 8 derived from Gustavsson et al. [25] and plotted against the linear dose region.

8 $$\:{D}_{\varDelta\:}^{95\%}=\:{k}_{p}\sqrt{2}\frac{\partial\:{N}_{CT}}{\partial\:D}u\left({N}_{CT}\right)$$

Where $\:{k}_{p}\sqrt{2}$ = 2.77 and u(N_CT_) is the standard uncertainty in the determination of N_CT_, calculated from Eq. 9 [26].

9 $$\:u\left({N}_{CT}\right)=\frac{{S}_{{N}_{CT}}}{\sqrt{n}}$$

Where n is the number of pixels in the region of interest and S_Nct_ is the standard deviation of N_CT_.

## Results

### Composition optimisation

#### Optimal cosolvent determination

**Fig. 2:**
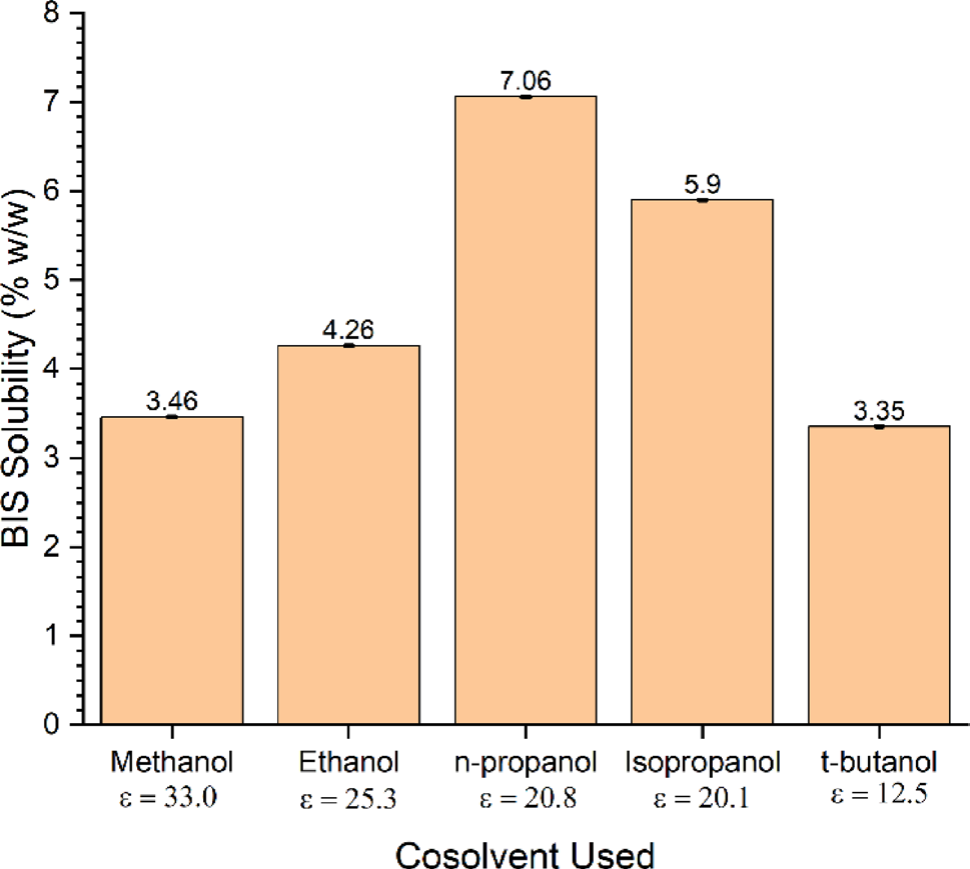
BIS solubility in various 3:7 cosolvent-to-water ratio solutions. Note ε is the relative permittivity, a relative measure of a compound’s polarity. Error bars included but are not visible

The first stage of the study involved investigating various co-solvent: water combinations to achieve improved BIS solubilisation. Figure 2 illustrates BIS solubility for a range of 3:7 co-solvent: water solutions. The solubility of BIS increased for the series of co-solvents methanol, ethanol, and propanol, which appeared to correspond with decreasing co-solvent polarity. However, BIS solubility in the isopropanol solution was lower than in n-propanol, despite the similar polarity of the two co-solvents. Furthermore, the solubility of BIS in the t-butanol solution was lower than isopropanol despite the lower polarity of the former. Based on these results, the most effective cosolvent for BIS dissolution was observed to be n-propanol, being able to dissolve 7.06 ± 6.13 × 10^− 3^% w/w of BIS at 18.5 °C.

#### Optimising the cosolvent ratio

**Fig. 3:**
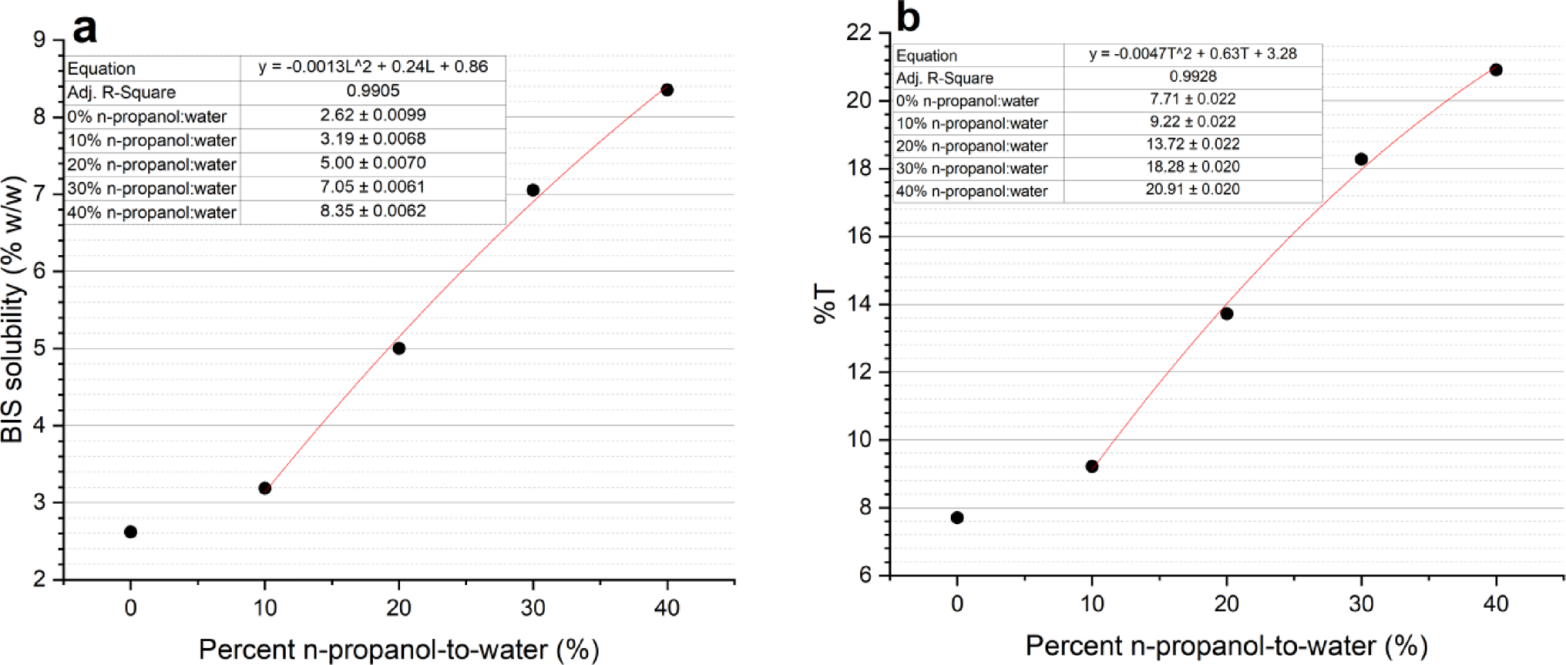
(**a**) Crosslinker solubility in solutions for varying percent n-propanol-to-water. (**b**) Maximum %T for varying percent n-propanol-to-water at 30%C. Errors bar included but are not visible

The next step was to determine the optimum n-propanol: water ratio for BIS solubility. The solubility of BIS in solutions of various n-propanol-to-water ratios can be seen in Fig. 3a. A second-degree polynomial of − 0.0013L^2^ + 0.24 L + 0.86 was found to best describe the BIS solubility limit per percent n-propanol-to-water (from 10 to 40%), with an adjusted R^2^ equal to 0.99. The maximum addition of %T possible for each aforementioned ratio is shown in Fig. 3b. A second-degree polynomial of y = -0.0047T^2^ + 0.63T + 3.28 was calculated to best (R^2^ = 0.99) describe the increase in %T with percent n-propanol-to-water (from 10 to 40%). Using the maximum %T calculations, chemically optimised samples for each n-propanol-to-water ratio were determined (Table 1).

**Table 1:**
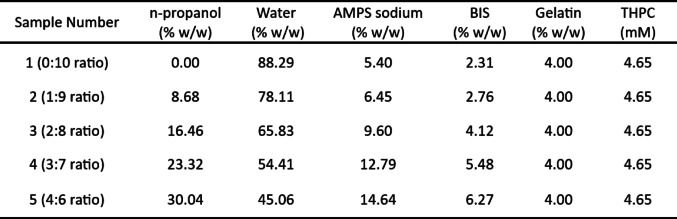
Chemical Distribution for Each Optimised Sample

**Fig. 4:**
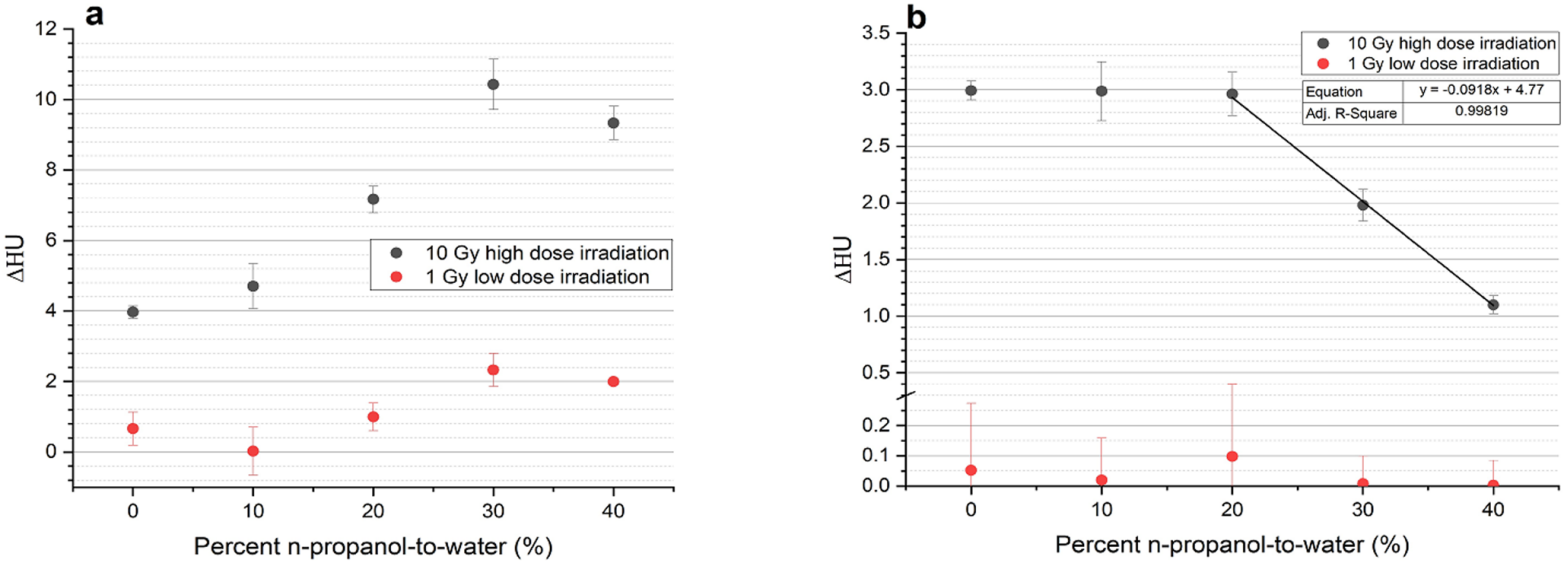
(**a**) ΔHU of optimised gel recipes for high and lose dose irradiations. (**b**) Changes in ΔHU of gel samples with varying percent n-propanol-to-water but constant %T, showing the direct effect the cosolvent addition has on the gel system

Figure 4a shows the ΔHU observed for optimised gel recipes with varying n-propanol-to-water ratios. It is noted that for both high dose and low dose irradiations, there was a linear increase (adjusted R^2^ = 0.975 and 0.977 respectively) in ΔHU for samples consisting of between 10 and 30% n-propanol-to-water. The 30% n-propanol-to-water sample (sample 4 with 18.3%T, 30%C) was observed to be the optimal formulation (referred to in subsequent text as PASSAG-N), with a 163.1 and 250.0% increase in ΔHU for high and low dose irradiations, respectively, relative to the n-propanol free sample. Despite an additional 2.64%T increase from sample 4 to 5, a 9.67 and 2.60% ΔHU decrease was observed in the high and low dose irradiations respectively.

Figure 4b shows the direct effect n-propanol has on ΔHU, with all gel samples having the same %T. For the high dose irradiation, a negative, linear correlation (adjusted R^2^ = 0.998) between ΔHU and percent n-propanol-to-water was exhibited for percentages larger than 20%. A 62.9% decrease in ΔHU was observed from 20 to 40% n-propanol-to-water. Similar trends were observed in the low dose region; however, a very low signal-to-noise ratio rendered these observations highly inaccurate.

### Theoretical tissue equivalency of the gel

**Fig. 5:**
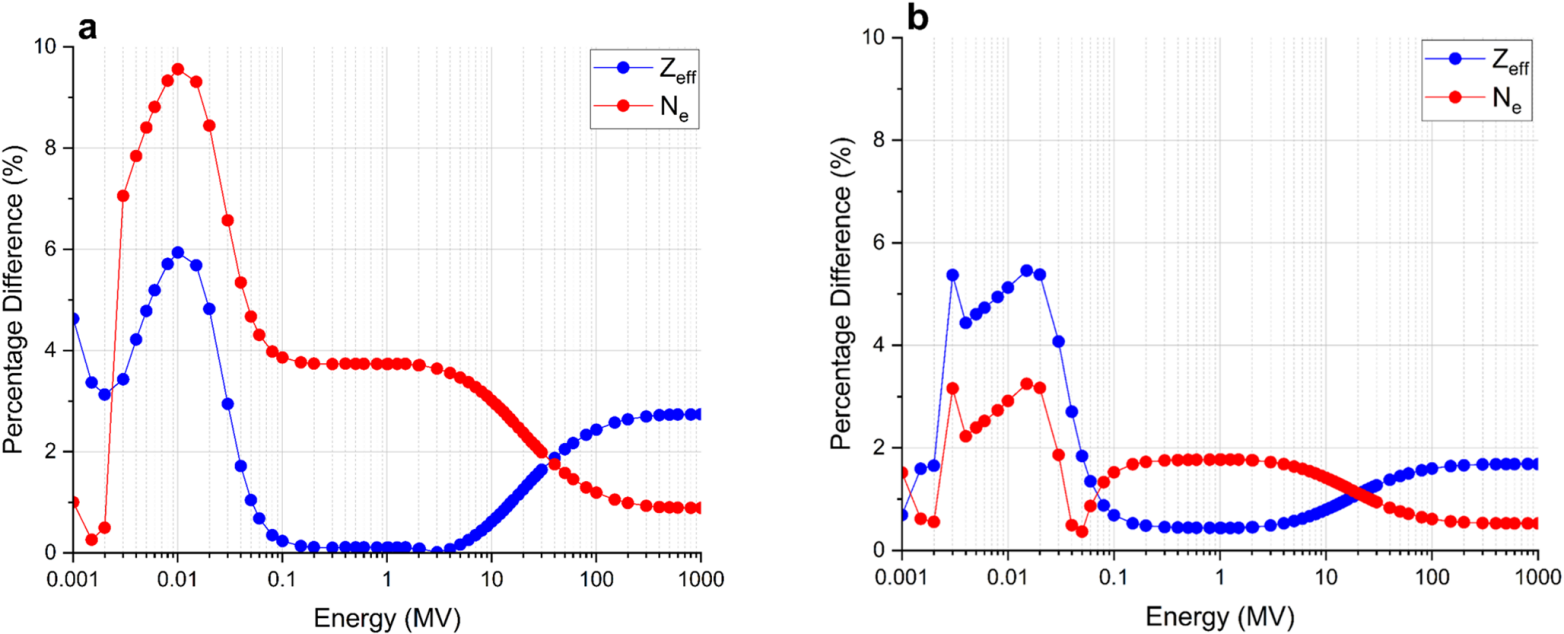
(**a**) Percentage difference in Z_eff_ and N_e_ between PASSAG-N and water. (**b**) Percentage difference in Z_eff_ and N_e_ between PASSAG-N and soft tissue. Lines added to help aid to viewer

The elemental composition by weight of the optimised PASSAG-N formulation along with soft tissue, water, and standard PASSAG is presented in Table 2. Percentage differences in Z_eff_ and N_e_ between PASSAG-N and water are shown in Fig. 5a, with the difference between PASSAG-N and soft tissue shown in Fig. 5b. There was a maximum 1.15 and 3.37% difference between Z_eff_ and N_e_ respectively, when comparing the gel to water in a 6–18 MV energy range. The gel was calculated to be closer to tissue equivalency with maximum differences in Z_eff_ and N_e_ of 1.05 and 1.59% respectively, for the same energy range.

**Table 2:**
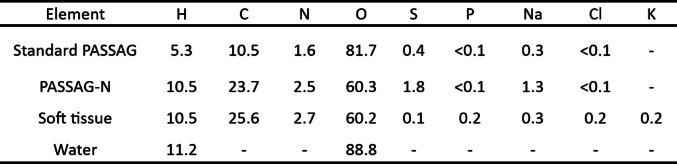
Elemental Composition by Weight of Optimised PASSAG-N, Soft Tissue, Water and Standard PASSAG

Note soft tissue composition data taken from ICRU Report 46 [24]

**Fig. 6:**
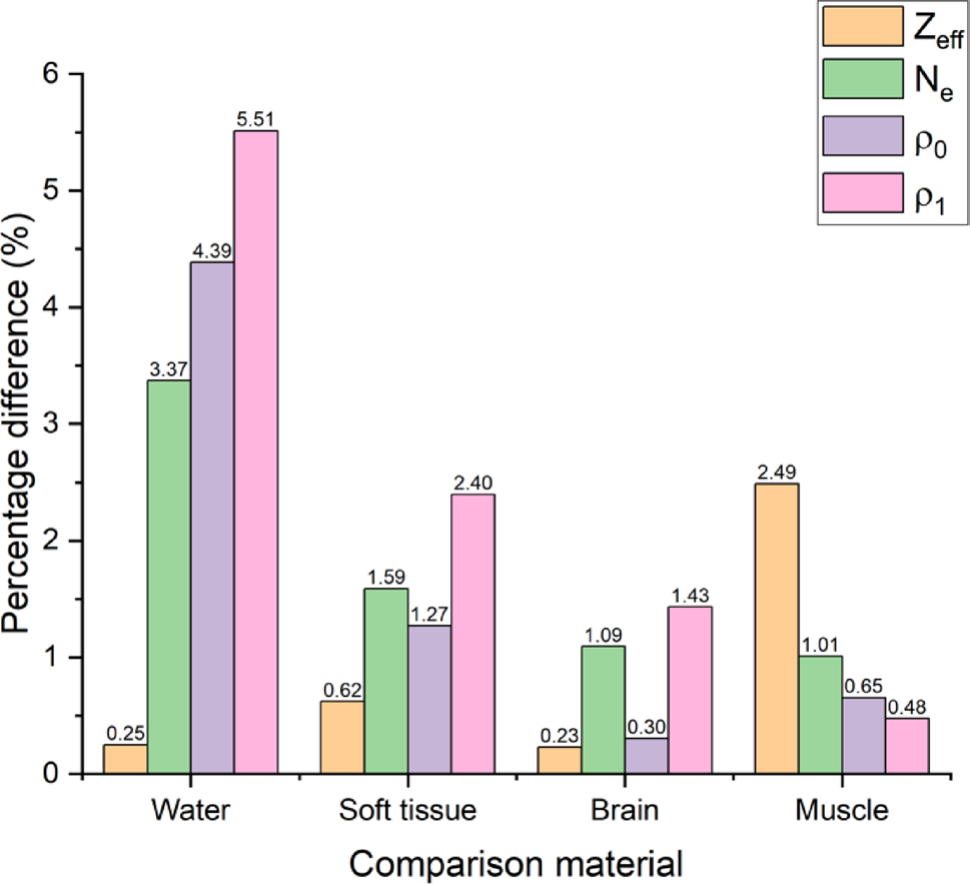
Percentage differences associated with Z_eff_, N_e_, ρ_0_, and ρ_1_ when comparing PASSAG-N to water and various tissue types at 6 MV. Note brain composition consisted of a 1:1 ratio of white and grey matter

The mass density of the unirradiated, optimised gel formula was determined to be 1.043 ± 1.5 × 10^− 2^ g/ml at 18.5 °C. Figure 6 shows the percentage differences associated with Z_eff_, N_e_, ρ_0_, and ρ_1_ when comparing PASSAG-N to water and various tissue types at 6 MV.

It was demonstrated that unirradiated PASSAG-N was theoretically equivalent to all simulated tissue types, with Z_eff_, N_e_ and ρ_0_ less than 2.5% different for all tissue types. Of the tissue types tested, the gel showed the most similarities to brain tissue, with the largest observed difference of 1.09% relating to calculated electron density. The density of PASSAG-N irradiated to 12 Gy was calculated to be 1.055 ± 1.7 × 10^− 2^ g/ml, a density increase of 1.15%. Differences between ρ_1_ and all simulated tissue types remained below 2.5%, with soft tissue exhibiting the largest difference of 2.40%.

### Sensitivity, dose resolution and dose range determination

**Fig. 7:**
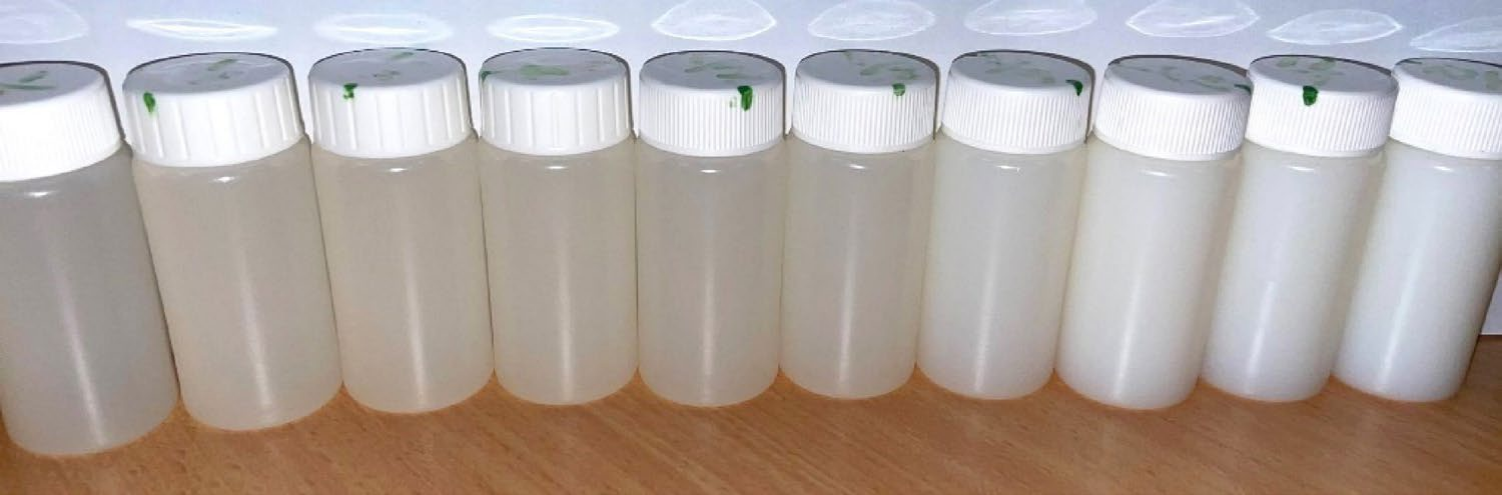
Visual change of PASSAG-N gel upon irradiation from 0–15 Gy. It is noted that the optical contrast and transparency was limited by the opaque plastic vials

**Fig. 8:**
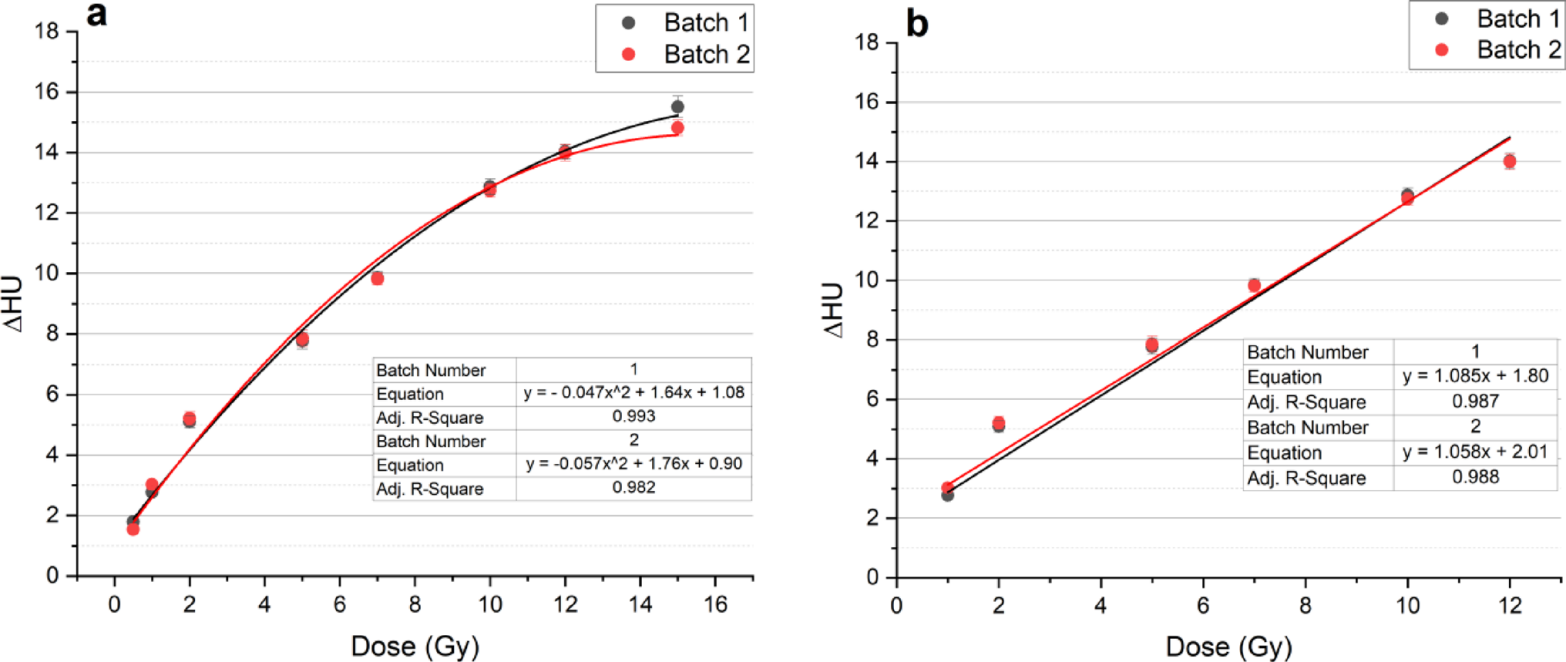
(**a**) PASSAG-N second-degree polynomial dose response over a 0.5–15 Gy region. (**b**) PASSAG-N linear dose response over a 1–12 Gy region

**Fig. 9:**
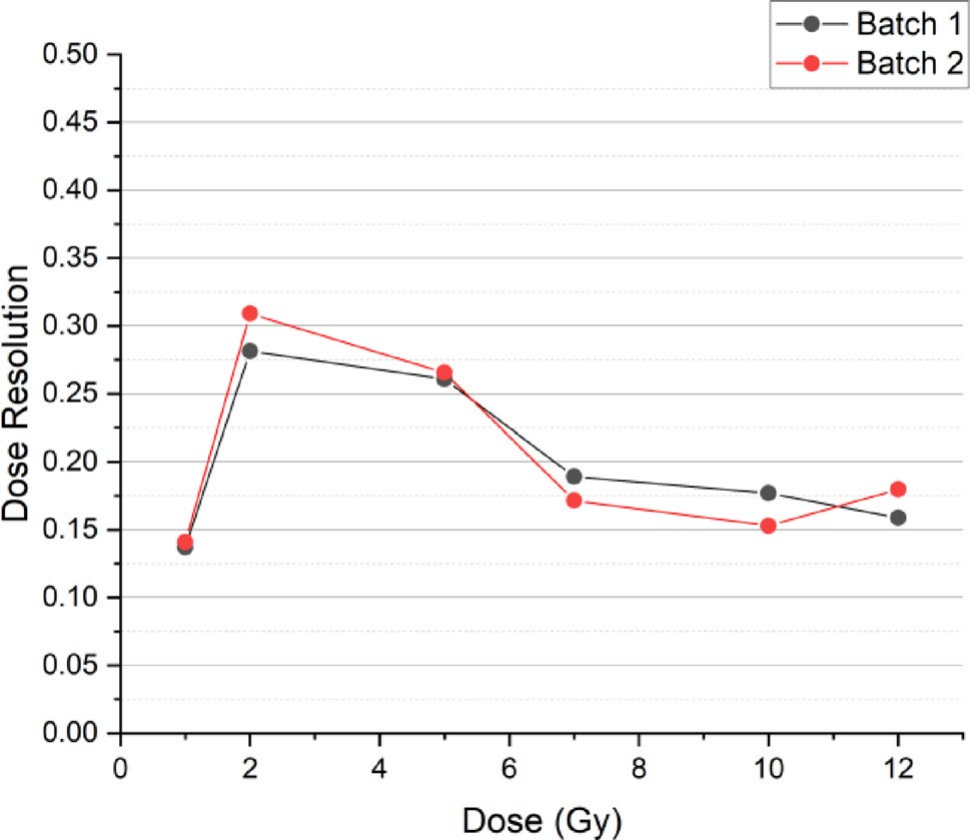
PASSAG-N dose resolution over the quasi-linear 1–12 Gy region. Lines added to help aid to viewer

Figure 7 shows the visual change in PASSAG-N over a 0–15 Gy region. The dose-response over the 0.5–15 Gy region described by a second-degree polynomial is illustrated in Fig. 8a. A quasi-linear regression over a 1–12 Gy region was fitted and shown in Fig. 8b. Error bars represent the standard deviation in mean pixel value taken from the centre of each vial. Excellent adjusted R^2^ values were observed in both polynomial and linear fits for both batches with values ranging between 0.982 and 0.993. Sensitivity for batch 1 and 2 were determined to be 1.085 ± 0.062 and 1.058 ± 0.052 HU Gy^− 1^ respectively. Figure 9 shows the dose resolution of PASSAG-N plotted across the quasi-linear dose range. Both gel batches showed reproducible dose resolutions, with values ≤ 0.31 Gy from 1 to 12 Gy. The highest dose resolution was observed at the 2 Gy irradiation point.

## Discussion

### Composition optimisation

#### Optimal cosolvent determination

Due to the homopolymer of AMPS being soluble in water [27], addition of a crosslinker is required to form a highly structured polymer network which can precipitate from solution, allowing dosimetric information to be read by an X-ray CT scanner [9]. The sparingly low solubility of BIS in water is one of the main limitations when attempting to increase BIS-based PGD sensitivity. Complete BIS dissolution is essential as any undissolved particulates can lead to an uneven distribution of crosslinker, resulting in dose distribution inaccuracies [9]. Cosolvents chosen for this experiment had to match a variety of criteria; a moderate polarity index to promote effective BIS dissolution, water-miscible at concentrations of up to 40% w/w, minimal-to-no net negative impact on the gel’s tissue equivalency, not significantly decreasing the pH of the solution as this would encourage autopolymerisation of AMPS [27, 28], relatively non-toxic, and low cost. The limited water solubility of BIS suggested that the crosslinker required a solvent with a lower relative permittivity than that of water (ε = 80.1) [29]. This was evident by the rising solubility when using co-solvents with larger hydrophobic carbon chains.

The relatively larger increase in BIS solubility when using both isopropanol and n-propanol solutions suggested that the compounds contained an optimal ratio of polar and non-polar functional groups. The hydrophilic polar groups ensured water miscibility whilst the hydrophobic non-polar groups disrupted the hydrogen-bonded network of the water molecules, reducing the interfacial tension between the water solution and solute [30]. A much larger increase in BIS solubility of 19.7% was observed when switching from isopropanol to n-propanol, despite their similar permittivity. An increase was also observed by Koeva et al.; however, it is noted that their experiment was conducted at 50 ^o^C as opposed to room temperature [9]. Additionally, no cloudiness was observed with the addition of n-propanol, contradictory to a previous report [9], which is hypothesised to result from the solubility behaviour of NIPAM and AMPS sodium salt in an n-propanol-water mixture.

Such a large difference in solubility was attributed to structural differences and steric effects. Isopropanol is a branched secondary alcohol, having two methyl groups in close proximity to the alcohol functional group. These methyl groups shield the hydrophilic alcohol group, resulting in steric hindrance [31, 32]. This reduces the hydrophilic group’s accessibility to partake in hydrogen bonding with water and the crosslinker, making it less energetically favourable to do so [31, 32]. The steric hindrance is more pronounced for the t-butanol, where three methyl groups shield the active site, which resulted in the significant reduction in BIS solubility.

#### Optimising the cosolvent ratio

The 2.62% w/w BIS solubility in pure water calculated using the gravimetric method was comparable to the 2–3% w/w noted by various authors, providing validation of the method used [9, 33].

A simple way to maximise sensitivity is to maximise %T, however, an optimal %C ratio must be maintained. Jirasek and Duzenli [34], determined that for a polyacrylamide gel, a 30%C gel was 1.8 times more sensitive than a 50%C gel in a 0–5 Gy dose region. Higher %C resulted in more prominent loops and knots in the polymer network structure, which slowed the rate of reaction and created a less sensitive gel [34]. The use of n-propanol as a cosolvent was observed to increase the solubility of the crosslinker which in turn made it possible for a higher %T gel to be manufactured.

The introduction of n-propanol as a cosolvent exerted a dual influence on the polymerisation-induced density changes. It had an indirect positive contribution through its ability to increase BIS solubility, allowing for formulations with notably higher %T; as well as a direct negative contribution when incorporated at n-propanol-to-water percentages larger than 20%.

The increase in %T resulted in more readily available monomer (and crosslinker) to be utilised for polymerisation. As seen in Fig. 4a this was beneficial in both low and high dose irradiations. For the high dose irradiations, gels with lower percent n-propanol-to-water (and therefore lower maximum %T) showed a limited ΔHU as opposed to the higher percent n-propanol-to-water gels. This suggests that there was dose-response saturation due to monomer depletion, with not enough monomers being available to undertake polymerisation as irradiation continued. Increases in %T through higher n-propanol-to-water ratios allowed for sufficient monomer to be available even at higher dose irradiations. For low dose irradiations, despite sufficient amounts of monomer present in all gel batches, increases in n-propanol-to-water ratios still resulted in increases in ΔHU. This is believed to have occurred due to the increased concentration of monomer resulting in a higher rate of polymerisation, as described in Eq. 10.

10 $$\:-\frac{d\left[M\right]}{dt}={k}_{p}{k}_{t}^{-0.5}{P}^{0.5}{G}_{M}^{0.5}{\left[M\right]}^{1.5}{\left(1+\frac{{G}_{s}\left[S\right]}{{G}_{M}\left[M\right]}\right)}^{0.5}$$

In this equation [S] and [M] are the solvent and monomer concentrations, respectively; k_p_ and k_t_ are the propagation and termination rates, respectively; G_M_ and G_S_ are the radiation yields of radical formation on monomer and solvent, respectively; and P is the dose rate [35].

The considerable decrease in ΔHU observed in Fig. 4b was likely attributable to two effects: (1) higher concentrations of n-propanol involved in chain transfer reactions reduced propagation, which increased the ratio of termination to propagation reactions and reduced the extent of crosslinking [35]. (2) a higher n-propanol-to-water ratio also led to greater penetration of solvent molecules into the polymer matrix, resulting in polymer swelling and a local density decrease [36].

From Fig. 4a, it is apparent that for concentrations ≤ 30% n-propanol-to-water, the positive contribution associated with n-propanol addition outweighed the negative contributions. As such, it was determined that the composition of sample 4 was the optimised PASSAG-N formulation to continue testing. This was in accordance with similar studies, which determined a 3:7 cosolvent-to-water ratio to be optimal [9]. The magnitude of both positive and negative contributions to ΔHU associated with cosolvent addition is likely to vary depending on the chemical used, and therefore it is recommended to conduct similar investigations when introducing new cosolvents. A previous investigation into enhancing PGD sensitivity using cosolvents showed that for NIPAM-based dosimeters, when both formulated at 10%T 50%C, gels made with isopropanol were more sensitive than those made with n-propanol [37]. This was speculated to be due to isopropanol acting as a chain-transfer agent in NIPAM polymerisation [37]. However, if the additional 19.7% BIS solubility afforded by n-propanol was utilised, it is theorised that a more sensitive dosimeter could have been created, with a maximum of 18.3%T compared to 15.7%T when using isopropanol (at a 30%C, 3:7 cosolvent-to-water ratio).

### Theoretical tissue equivalency of the gel

As more AMPS sodium salt and BIS are introduced into the system, the tissue equivalency of the gel decreases. Therefore, it is important to reaffirm the tissue equivalency of any newly optimised gel system. Unirradiated, optimised PASSAG-N showed good theoretical equivalency to water and all tissue types tested, with greater equivalency observed in the latter. Exceptionally low percentage differences were observed when compared to brain tissue, suggesting that PASSAG-N could make an excellent phantom for stereotactic radiosurgery patient specific quality assurance. With regards to soft tissue equivalence, the addition of n-propanol introduced a large portion of carbon atoms into the dosimeter, resolving the carbon deficiency/oxygen surplus observed in standard PASSAG and other PGDs [11, 38]. This removed the requirement to approximate soft tissue equivalency by summing carbon and oxygen content based on their cross-section equivalency [38]. Radiation-induced polymerisation leads to a local change in electron density due to the expulsion of water from polymer clusters, along with small decrease in PGD volume (< 1%), thereby resulting in an increased gel density [20, 39, 40]. The calculated ρ_1_ values deviated further from tissue densities, except in the case of muscle. Additionally, it is noted that the dosimeter showed promising application in neutron depth-dose measurements for both the keV and MeV range, with hydrogen, carbon, nitrogen, and oxygen content, along with mass density all within ± 10% of soft tissue, meeting requirements put forth in ICRU report 44 [41]. Large variations in percentage difference in both Z_eff_ and N_e_ from 3 to 40 keV can be attributed to the relatively large amount of sulphur present in the gel.

### Sensitivity, dose resolution and dose range determination

Dosimetric characteristics of the optimised PASSAG-N gel showed a clinically appropriate dose range of 0–15 Gy, with an excellent linear response over a 1–12 Gy region, making it ideal for stereotactic radiosurgery and other highly complex radiotherapy techniques. Preliminary testing on an unoptimized PASSAG showed only a minor change in HU from 15 to 50 Gy. Noting the rapid consumption of BIS relative to its monomer counterpart, this drop-off in sensitivity in the high dose region was likely due to a limited amount of BIS remaining for crosslinking, especially considering the 30%C formulation [34], resulting in linear AMPS polymers that could not effectively precipitate out of solution. PASSAG-N exhibited an average sensitivity of 1.072 ± 0.041 HU Gy^− 1^– approximately 1.3 times higher than PAG [5], which is anoxic and requires a complex manufacturing process; 1.3 times higher than MAGAT, which has been shown to be significantly dose rate dependent [13]; 1.5–3.5 times higher than PAGAT [13, 42]; 3.7 times higher than unoptimised NIPAM [9]; and 1.2–2.7 times higher than optimised NIPAM [9, 10]– all whilst utilising a monomer that is non-toxic relative to the formulations listed above, an important consideration for future clinical implementation. The dose resolution exhibited by PASSAG-N is comparable to other formulations imaged using X-ray CT. For optimised NIPAM, Johnston et al., observed a dose resolution ranging between 0.2 and 0.5 Gy for a 0–20 absorbed dose range [43]. Significant differences in dose response have been observed between small and large volume PGDs [44]. Hypotheses for these discrepancies include differences in cooling rate and temperature uniformity between gel volumes [45], along with physiochemical changes related to an oxygen-antioxidant imbalance [46]. Further tests using large-volume PASSAG-N dosimeters are required to determine the extent of any discrepancy between the small-volume dosimeters used in this study and those intended for clinical application.

## Conclusion

In this study, the sensitivity of a pre-established PASSAG polymer gel dosimeter was optimised, using a scientific method-based approach, to increase feasibility for clinical implementation. This is the first investigation into X-ray CT imaged PASSAG gels to our knowledge, utilising an imaging method that would be best suited for a busy public hospital. An increase in sensitivity was accomplished through the addition of n-propanol, promoting further BIS dissolution, and thereby allowing for a higher total monomer concentration. A quantitative gravimetric method was shown to be adequate in determining BIS solubility with cosolvent addition. An 18.3%T 30%C PASSAG-N gel was determined to be the optimal composition, exhibiting significantly larger changes in HU relative to the n-propanol-free PASSAG formulation. The optimised formulation was found to be theoretically equivalent to water, soft tissue, brain, and muscle over a large clinical energy range. Overall, the optimised dosimeter exhibited sensitivities higher than other published formulations imaged with X-ray CT, whilst exhibiting comparable dose resolution, demonstrating the importance of optimisation. Further testing with large volume dosimeters is required to develop a better indication of clinical feasibility.
